# Beyond the usual suspects: rethinking post-stroke immunosuppression

**DOI:** 10.3389/fimmu.2026.1824573

**Published:** 2026-05-13

**Authors:** Laia Ascaso-Vidal, Alba Simats, David Brea

**Affiliations:** 1 Department of Neuroscience and Experimental Therapeutics, Instituto de Investigaciones Biomédicas de Barcelona(IIBB), Consejo Superior de Investigaciones Científicas (CSIC), Barcelona, Spain; 2 Programa de Doctorado en Biomedicina, University of Barcelona, Barcelona, Spain

**Keywords:** ischemic stroke, lymphopenia, peripheral immunodepression, post-stroke infections, stroke-induced immunosuppression

## Abstract

Ischemic stroke extends far beyond the hyperacute vascular event. In addition to the immediate ischemic injury, patients frequently develop systemic complications that significantly influence outcome. Among these, a biphasic immune response has emerged as a central feature: an early inflammatory reaction followed by a state of peripheral immunosuppression. This immunosuppressive phase has been consistently associated with increased susceptibility to post-stroke infections, particularly pneumonia, thereby contributing to morbidity and mortality. Multiple mechanisms have been implicated in the development of stroke-induced immunosuppression, including activation of the autonomic nervous system and the hypothalamic-pituitary-adrenal axis, the release of damage-associated molecular patterns (DAMPs), reprogramming of bone marrow hematopoiesis, and peripheral neutrophil activation with downstream effects on lymphocyte survival. While these pathways are often studied in isolation, accumulating evidence suggests that they may interact within a coordinated neuroimmune network. In this review, we not only summarize the current understanding of the mechanisms underlying post-stroke immunosuppression but also explore how these processes may converge and influence one another. Finally, we discuss the unresolved question of whether this immunosuppressive state represents an adaptive response aimed at protecting the injured brain or a maladaptive bystander consequence of disrupted neuroimmune homeostasis.

## 1 Introduction: ischemic stroke, pathophysiology and immune response

Acute stroke is defined as the sudden onset of focal neurological deficits in a vascular territory affecting the brain, retina, or spinal cord due to underlying cerebrovascular disease (1). Stroke remains a major global health challenge, with 12.2 million new cases annually, 101 million people currently living after stroke, and approximately one in four individuals over the age of 25 expected to experience a stroke during their lifetime (2). It is the second leading cause of death worldwide and a major contributor to long-term disability and economic burden (2). Strokes are broadly categorized as ischemic and hemorrhagic. Ischemic stroke accounts for approximately 85% of incident strokes globally and will therefore be the focus of this review (2, 3).

Ischemic stroke results from thrombotic or embolic occlusion of a cerebral artery, leading to impaired blood flow and, consequently, to oxygen and glucose deprivation in the affected brain region. This energetic failure rapidly disrupts ionic homeostasis, promoting glutamate excitotoxicity, oxidative stress, and inflammation that ultimately result in infarction if perfusion is not restored in time. During this process, the infarct core is surrounded by the ischemic penumbra, a metabolically vulnerable region that may still be salvaged by timely reperfusion (4). Although recanalization therapies such as intravenous thrombolysis and mechanical thrombectomy can improve outcome, therapeutic options remain limited and many patients still develop substantial secondary injury.

Beyond the acute vascular event, stroke also triggers a complex inflammatory response involving both the central nervous system (CNS) and the periphery. Cellular injury leads to the release of damage-associated molecular patterns (DAMPs), including ATP, HMGB1, heat shock proteins, peroxiredoxins, mitochondrial-derived peptides, and extracellular DNA, which activate resident glial cells and promote endothelial dysfunction and blood-brain barrier breakdown (5, 6). These events facilitate the recruitment of circulating immune cells and amplify innate and adaptive immune responses within the ischemic brain (7). In experimental models, neuroinflammation is characterized by early microglial activation followed by infiltration of neutrophils, monocytes/macrophages, NK cells, T cells, and B cells (7–10). In humans, neutrophil accumulation is mainly observed in the ischemic core during the first days after stroke, whereas microglial activation is more prominent in the surrounding penumbra (11). Together, these coordinated innate and adaptive immune responses constitute the neuroinflammatory phase of ischemic stroke.

However, stroke is not solely associated with neuroinflammation. Beyond the acute ischemic injury, stroke induces systemic immune alterations that extend far beyond the CNS. Among these, a subsequent phase of peripheral immunosuppression, commonly referred to as stroke-induced immunosuppression (SIIS), has been consistently observed and is associated with increased susceptibility to infection and poorer clinical outcome (5). Understanding how ischemic injury transitions from acute neuroinflammation to systemic immune dysfunction is therefore essential to clarify the mechanisms, biological significance, and therapeutic implications of post-stroke immunosuppression.

## 2 Peripheral immune alterations after stroke

Early after stroke, in a short period of time after neuroinflammation, systemic immunodepression develops in parallel. This phenomenon, referred to as stroke-induced immunosuppression (SIIS), encompasses a series of processes that result in peripheral immune dysfunction following cerebral ischemia (12).

Immunosuppression associated with stroke was described as early as 1974 (13). However, this phenomenon is not exclusive to stroke. Similar patterns of immune depression have been reported in other life-threatening conditions, including polytrauma, major surgery, and central nervous system injuries such as traumatic brain injury (TBI) and spinal cord injury (SCI) (14–17).

The main manifestations of SIIS include lymphopenia (predominantly affecting CD4^+^ T cells), an increased neutrophil-to-lymphocyte ratio, decreased expression of antigen-presenting molecules (e.g., HLA-DR) on monocytes, and a shift from Type 1 (Th1) to Type 2 (Th2) helper T-cell responses. This shift is characterized by reduced production of pro-inflammatory cytokines such as IFN-γ and TNF-α and increased production of anti-inflammatory mediators, particularly IL-10 (18). In cases of extensive stroke, reductions in body weight and spleen weight have also been observed (19). This spleen atrophy has also been observed in patients after stroke (20, 21). In the case of the thymus, a similar decrease in thymus size has also been observed together with a significant decrease of T cells after stroke (21).

With regard to lymphopenia, numerous clinical and preclinical studies have reported decreased levels of B cells, CD4^+^ T cells, CD8^+^ T cells, γδ T cells, and natural killer (NK) cells following stroke. In murine models, increased apoptosis of lymphocytes in lymphoid organs, including the spleen and thymus, has been detected as early as 12 hours post-stroke (22). In patients, lymphopenia at 3 days after stroke has been independently associated with poor functional outcome at 3 months (23).

The Th1-to-Th2 shift reflects reduced production of Th1-derived pro-inflammatory cytokines-including IFN-γ, IL-2, IL-12, and TNF-α, and increased secretion of Th2-associated cytokines such as IL-4, IL-5, IL-10, and IL-13 (18). A decreased Th1/Th2 ratio has also been described in patients undergoing major surgery or trauma and has been associated with reduced survival (24–27).

Lymphoid organ alterations constitute an important component of stroke-induced immunosuppression. In particular, the spleen has emerged as a major peripheral immune organ affected after stroke. Experimental studies have shown that stroke alters splenic function, promotes the loss of splenic immune cells, and contributes to the infection-prone state observed after cerebral ischemia. In addition, a recent preclinical meta-analysis evaluating splenectomy in murine stroke models reported a significant reduction in infarct volume (28), further supporting the relevance of splenic responses in stroke pathophysiology. Alterations are also observed at the level of the bone marrow. Although the bone marrow primarily serves as the source of granulocytes and monocytes, approximately 1-2% of bone marrow mononuclear cells are CD4^+^ T cells (29, 30). Interestingly, experimental studies have reported an increase in CD4^+^ T-cell numbers in the bone marrow after stroke (29). Within this population, regulatory T cells (Tregs) are significantly elevated, as reflected by an increased Treg/CD4^+^ T-cell ratio at 3 and 7 days post-stroke (31). Additionally, murine studies have shown that B-cell development is impaired at the pro-B-cell stage in femoral bone marrow as early as 3 days after transient middle cerebral artery occlusion (tMCAO) (32).

In patients, post-stroke immunosuppression is further characterized by reduced peripheral blood lymphocyte counts and impaired T-cell and NK-cell activity, reinforcing the concept of systemic immune dysfunction following cerebral ischemia (33).

## 3 Clinical consequences of post-stroke immunosuppression: infections and outcome

Beyond the direct effects of brain injury, a substantial fraction of early morbidity and mortality after stroke is driven by medical complications, particularly infections. Early systematic studies already established that infections are common in the acute phase: a 2011 systematic review and meta-analysis reported an overall post-stroke infection rate of ~30%, with pooled rates of pneumonia and urinary tract infection (UTI) each around ~10% (34). More recent epidemiological data suggest that the *overall* prevalence of post-stroke infections may be decreasing. A 2024 systematic review including >32 million stroke patients reported a pooled prevalence of post-stroke infections of 9.14%, with pneumonia and UTI remaining the dominant entities (pneumonia 12.4% and UTI 8.31% in their pooled estimates, reflecting heterogeneity across definitions and cohorts) (35). Taken together, these data support that post-stroke infections are a reproducible and clinically relevant phenomenon, even if their reported incidence varies markedly across settings and eras.

Regarding microbiology, the etiological spectrum of stroke-associated pneumonia (SAP) remains imperfectly characterized, largely because microbiological confirmation is inconsistently pursued and diagnostic criteria are heterogeneous. A dedicated systematic review on microbiological etiologies of pneumonia complicating stroke highlighted that culture positivity rates vary widely (15%-88%) and that most pneumonia occurs within the first week after stroke (36). When pathogens are identified, recent large-scale synthesis points toward the commonest organisms reported were Klebsiella pneumoniae (8.5-41.5%), Acinetobacter baumannii (16.8%), Enterococcus faecalis (18.5%), Staphylococcus aureus (11.5-15%), Klebsiella oxotyca (12.9%), Proteus mirabilis (12.9%), Escherichia coli (1.9-11.2%), Pseudomonas aeruginosa (11.2%), and Enterobacter aerogenes (9.3%). A clinically concerning burden of multidrug-resistant organisms, including carbapenem-resistant *Klebsiella pneumoniae*, methicillin-resistant *Staphylococcus aureus*, and carbapenem-resistant *Acinetobacter baumannii* in some cohorts has also been identified (35). The pooled mortality rate of post-stroke infections was 15.91% (95% CI [8.24; 25.31]). The estimated mortality rates of pneumonia and UTI were 21.60% (95% CI [0.10; 62.26]) and 12.16% (95% CI [0.00; 40.31]) respectively. This variability and evolving resistance landscape complicate the design of broadly effective preventive strategies.

These observations motivated multiple clinical trials testing whether early preventive antibiotics can reduce infections and improve outcome. Early randomized studies were relatively small and yielded mixed results. The ESPIAS trial (2005) showed that prophylactic levofloxacin was not superior to standard care in preventing infections after acute stroke (37). The phase IIb PANTHERIS trial (2008) suggested that, although preventive moxifloxacin reduced infections after severe ischemic stroke, neurological outcome and survival were not significantly influenced by treatment with moxifloxacin (38). As the field moved into larger pragmatic trials, the clinical message became clearer: the PASS trial (2015) demonstrated that preventive ceftriaxone did not improve functional outcome at 3 months, despite effects on infection rates (39). In parallel, other large trials targeting high-risk subgroups (e.g., dysphagic patients) similarly failed to demonstrate convincing clinical benefit of prophylactic antibiotics on the outcomes that matter most (40).

Importantly, detailed analyzes from these trials and subsequent systematic reviews have highlighted that the reduction in overall infections observed with preventive antibiotic therapy is largely driven by decreases in urinary tract infections, whereas rates of pneumonia, the complication most consistently associated with poor functional outcome and mortality, are not significantly reduced (41, 42). This distinction may help explain why reductions in total infection rates have not translated into improved neurological recovery or survival.

Consistently, reviews summarizing the accumulated randomized evidence conclude that while preventive antibiotics can reduce *overall infections*, they do not improve functional outcome or reduce mortality, and therefore are not recommended as routine prophylaxis in unselected stroke patients.

This creates an important, and still unresolved, clinical controversy: do post-stroke infections causally worsen outcome, or are they primarily a marker of larger strokes and more severe neurological deficits? Observational evidence supports an association between pneumonia and both unfavorable outcome and death, and meta-analytic data have identified pneumonia as an independent risk factor (34). Yet the neutral outcome results of antibiotic prophylaxis trials argue that simply lowering infection incidence (often driven by reductions in certain infection categories) is not sufficient to translate into better neurological recovery. Potential explanations include heterogeneity in infection definitions, incomplete prevention of SAP driven by aspiration and non-bacterial mechanisms, suboptimal timing/targeting of antibiotics, and the possibility that infection is tightly intertwined with stroke severity and SIIS rather than being a fully independent determinant. Importantly, even under this uncertainty, infections remain clinically significant complications and multiple datasets link infection susceptibility to SIIS phenotypes-e.g., early IL-10 elevations and monocytosis or reduced monocytic HLA-DR expression associated with early infection risk, and deeper T-cell impairment associated with infectious complications (38, 43).

## 4 Mechanisms of post-stroke immunosuppression

### 4.1 The sympathetic nervous system

**Figure 1:**
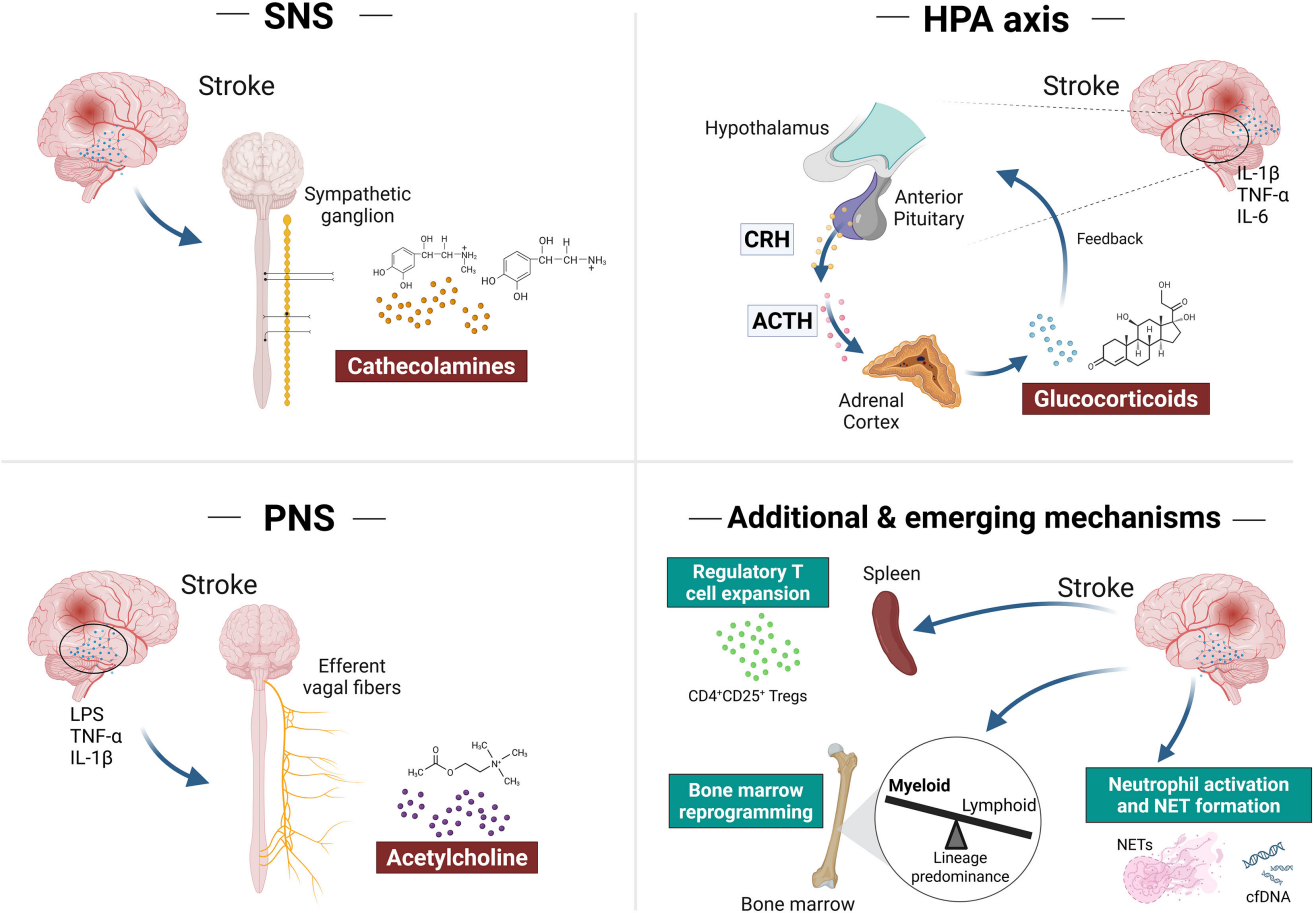
Mechanisms of post-stroke immunosuppression. Stroke induces the activation of the sympathetic nervous system (SNS; top left panel), the hypothalamic-pituitary adrenal (HPA) axis (top right panel) and parasympathetic nervous system (PNS; bottom left panel) and other additional and emerging mechanisms (bottom right panel). ACTH, adrenocorticotropic hormone; cfDNA, cell-free DNA; CRH, corticotropin-releasing hormone; HPA, hypothalamic-pituitary adrenal; IL-1β, interleukin-1β; IL-6, interleukin-6; LPS, lipopolysaccharide; NETs, neutrophil extracellular traps; PNS, parasympathetic nervous system; SNS, sympathetic nervous system; TNF-α, tumor necrosis factor-α.

One of the most extensively proposed mechanisms mediating SIIS is activation of the sympathetic nervous system (SNS). The SNS originates in brainstem nuclei, including the locus coeruleus and the rostral ventrolateral medulla, from which preganglionic cholinergic efferent fibers project to paravertebral and prevertebral ganglia. From these ganglia, postganglionic sympathetic fibers innervate peripheral organs and release norepinephrine at target tissues. Beyond its well-known physiological effects associated with the “fight-or-flight” response, such as increased heart rate, vascular tone, respiratory rate, gastrointestinal modulation, and heightened arousal, the SNS exerts profound immunomodulatory effects (33) (See **Figure 1**).

Catecholamines released upon SNS activation interact directly with immune cells. All major immune cell populations, including lymphocytes, monocytes, macrophages, granulocytes, and natural killer cells, express adrenergic receptors, predominantly β1- and β2-adrenergic receptor (33, 44–47). Engagement of these receptors increases intracellular cyclic AMP levels and activates protein kinase A signaling pathways, ultimately leading to modulation of gene transcription. Functionally, this signaling cascade suppresses immune cell activation, promotes a shift from Th1- to Th2-type responses, reduces production of pro-inflammatory mediators such as TNF-α, IL-1β, IL-12, IFN-γ, and nitric oxide, and favors the release of anti-inflammatory cytokines including IL-6 and IL-10 (33).

Importantly, SNS activation occurs very early after stroke. Plasma catecholamine metabolites, such as metanephrine, have been reported to rise as early as 2 hours post-stroke and remain elevated for several days (19). Similarly, systemic norepinephrine and epinephrine levels increase within hours after ischemia, indicating rapid engagement of the sympathetic axis (48). Both experimental and clinical data suggest that the magnitude of catecholamine elevation correlates with stroke severity, supporting the notion that SNS activation scales with infarct size (49). In patients, elevated circulating catecholamines and their metabolites have been documented following acute ischemic stroke, particularly in individuals who subsequently develop infectious complications (50, 51). However, not all clinical studies have consistently observed significant increases, reflecting heterogeneity in patient populations, timing of sampling, and stroke characteristics (52, 53). Nevertheless, the overall body of evidence supports early and robust activation of the SNS as a hallmark of the acute post-stroke response.

A substantial body of experimental evidence supporting the role of the SNS in SIIS derives from systemic intervention studies in animal models. Pharmacological ablation of sympathetic terminals using 6-hydroxydopamine (6-OHDA) has been widely employed to assess the contribution of SNS signaling. 6-OHDA selectively enters noradrenergic neurons and destroys sympathetic nerve terminals, leading to depletion of tissue catecholamines. In murine models of stroke, 6-OHDA treatment prevented spontaneous bacterial infections in blood and lungs and restored IFN-γ production (22). It was also sufficient to reverse post-stroke alterations in hepatic invariant natural killer T (iNKT) cells (54). In rat models, chemical sympathectomy reduced early systemic inflammatory markers and improved long-term neurological outcome, whereas treatment with labetalol did not produce comparable effects (55). These findings collectively indicate that intact sympathetic signaling contributes to post-stroke immune dysfunction and infection susceptibility, however chemical sympathectomy does not allow for analysis of receptor-specific effects.

Pharmacological blockade of adrenergic receptors has provided further mechanistic insight. In rodent models, the non-selective β-adrenergic antagonist propranolol reduced infarct size in rats (56) and prevented the reduction in peripheral blood lymphocyte counts observed after stroke in mice (22). Propranolol normalized splenocyte apoptosis to levels comparable to sham-operated animals, reduced bacteremia and pneumonia, and improved survival after experimental stroke (22). In addition, propranolol treatment reverted the increase in CD4^+^CD25^+^FoxP3^+^ regulatory T cells observed in the bone marrow, suggesting that SNS activation contributes to Treg expansion (31). Selective targeting of β2-adrenergic receptors has yielded more complex results: genetic deletion of β2-AR or pharmacological inhibition with ICI118,551 reduced infarct size and inflammatory gene expression in some models (57, 58), yet other studies failed to observe significant effects on spleen weight or lymphocyte numbers (19), and spontaneous lung infection rates were similar in Adrb2^-^/^-^ mice and controls (48). These discrepancies suggest that β2-adrenergic signaling, while important, is unlikely to be the sole driver of post-stroke immune impairment.

The contribution of α-adrenergic signaling has also been explored. In rats, treatment with the selective α1-adrenergic antagonist prazosin, but not propranolol, reduced TNF-α levels and prevented post-stroke splenic shrinkage, suggesting that splenic contraction may partly result from adrenergic stimulation of the smooth muscle capsule (59). In contrast, studies in mice have attributed splenic atrophy primarily to extensive apoptosis of splenocytes, accounting for up to 90% of cell loss at 96 hours after MCAO, with a marked reduction in B-cell populations (60). Together, these findings indicate that the mechanisms underlying splenic morphological changes may differ between species and likely involve both neurogenic contraction and immune cell apoptosis (61).

Clinical data on β-blockers have been less supportive than preclinical studies. While several retrospective analyzes have reported associations between post-stroke β-blocker use and lower mortality and/or reduced rates of stroke-associated infections, often using pneumonia as the main clinical endpoint (62), other observational studies have found no benefit or even higher infection rates among β-blocker users (63). In fact, a meta-analysis comprising more than 100,00 patients concluded that β-blockers do not significantly improve stroke outcomes or lower post-stroke infections rates (64). A prospective study observed different effects in selective and non-selective β-blockers and found that non-selective β-blocker were associated with higher pneumonia and UTI infections, while β1-selective blocker was not (65). It seems that β-blocker selectivity could explain the discrepancies in the clinical data, however most study fail to report β-blocker specificity.

Regarding the discrepancies between the preclinical and clinical data, these different results could possibly be explained by the different doses, as the high doses of β-blockers used in animal studies cannot be used in humans where hypotension, bradycardia, and reduced cerebral perfusion are relevant concerns (18).

In summary, the precise contribution of the sympathetic nervous system to post-stroke immunosuppression remains incompletely defined. Although numerous experimental studies support a role for SNS activation, the overall body of evidence is marked by contradictory findings. Importantly, most available studies have relied on relatively broad and systemic approaches, such as chemical sympathectomy with 6-hydroxydopamine or the use of non-selective or partially selective adrenergic receptor antagonists. These strategies disrupt sympathetic signaling globally and do not allow discrimination between direct effects on immune cells and indirect effects mediated through hemodynamic, neuroendocrine, or organ-specific pathways.

### 4.2 The hypothalamic-pituitary-adrenal axis

Another major neuroendocrine pathway implicated in post-stroke immunosuppression is the hypothalamic-pituitary-adrenal (HPA) axis. Following cerebral ischemia, pro-inflammatory cytokines released in the brain, including IL-1β, TNF-α, and IL-6, stimulate neurons within the paraventricular nucleus (PVN) of the hypothalamus to produce corticotropin-releasing hormone (CRH). CRH is secreted into the hypophyseal portal circulation and binds to corticotropin-releasing hormone receptor 1 (CRHR1) in the anterior pituitary, inducing the release of adrenocorticotropic hormone (ACTH). ACTH subsequently stimulates glucocorticoid synthesis in the zona fasciculata of the adrenal cortex (33) (See **Figure 1**).

Glucocorticoids exert potent immunomodulatory effects. They suppress the production of pro-inflammatory mediators such as IL-1β, IL-6, IL-12, TNF-α, IFN-γ, prostaglandins, and nitric oxide, while promoting anti-inflammatory cytokines such as IL-10 and transforming growth factor-β (TGF-β). At the cellular level, glucocorticoids induce apoptosis in lymphocytes, impair antigen presentation, and reduce cytokine synthesis, thereby contributing to systemic immunosuppression. The shift from Th1 to Th2, however, is more likely to be SNS-mediated (22) as HPA primarily causes quantitative T cell loss rather than functional changes (19). Thus, current evidence suggests that HPA activation mainly affects T-cell abundance and survival, whereas functional polarization of T-cell responses appears to depend more strongly on sympathetic signaling. Finally, through classical negative feedback mechanisms, elevated glucocorticoid levels also suppress further CRH and ACTH release (33).

The HPA axis is activated early after stroke. Experimental studies have demonstrated that serum corticosterone levels increase within hours after cerebral ischemia and remain elevated for several days. Interestingly, work from Veltkamp and colleagues showed that sustained post-stroke glucocorticoid elevation does not necessarily depend on continued ACTH stimulation, suggesting that cytokines such as IL-6 and IL-1β may directly modulate adrenal function and contribute to prolonged hypercortisolemia (19).

In patients, elevated serum cortisol levels have been consistently observed after acute ischemic stroke (66). Increased cortisol concentrations correlate inversely with peripheral lymphocyte counts and have been associated with larger infarct volumes, poorer functional outcome, and increased mortality (23, 33). Both excessive and insufficient ACTH-cortisol responses appear to be linked to worse prognosis, indicating that dysregulation, rather than simple overactivation, of the HPA axis may be clinically relevant (33).

Preclinical studies further support a functional role of glucocorticoid signaling in post-stroke immune alterations. Treatment with the glucocorticoid receptor antagonist RU486 reduced infarct volume, improved survival, prevented lymphopenia, and attenuated splenic apoptosis, restoring splenocyte numbers to sham levels in experimental models (22, 67). Similarly, blockade of glucocorticoid signaling normalized spleen weight and monocyte function after stroke (19). In bone marrow, glucocorticoid signaling appears to mediate impaired B-cell development: CD19-specific glucocorticoid receptor knockout mice showed restoration of B-cell production after stroke, whereas sympathectomy or β-adrenergic blockade failed to rescue this phenotype, suggesting that HPA axis activation regulates B-cell suppression in this context (32, 68).

Additional experimental evidence indicates that modulation of the HPA axis can influence both brain injury and systemic immune responses. In diabetic mice, which exhibit chronic HPA axis hyperactivation and elevated corticosterone levels, stroke resulted in larger infarcts and increased expression of inflammatory mediators in the ischemic brain. Pharmacological inhibition of glucocorticoid synthesis with metyrapone reduced IL-6 expression and infarct size in these animals, suggesting that excessive HPA activation may exacerbate stroke pathology under certain metabolic conditions (69). Conversely, treatment with Astragaloside IV (ASIV), a compound shown to suppress CRH expression and reduce corticosterone levels, attenuated splenic atrophy, preserved lymphocyte populations, and reduced peripheral immunosuppression in MCAO models, further implicating HPA activation as a mechanistic driver of post-ischemic immune dysfunction (70).

However, the relative contribution of the HPA axis compared with sympathetic activation remains debated. In the seminal study by Prass et al., blockade of the sympathetic nervous system, but not inhibition of the HPA axis, prevented spontaneous bacterial infections after experimental stroke and restored IFN-γ responses, suggesting that catecholamine-mediated mechanisms may play a dominant role in early antibacterial immune impairment (22). These findings indicate that the HPA axis is likely one component of a broader neuroendocrine network, interacting with but not fully overlapping the effects of sympathetic activation.

Overall, substantial experimental and clinical evidence supports activation of the HPA axis after stroke and implicates glucocorticoids in mediating lymphocyte apoptosis, bone marrow suppression, and altered cytokine balance. Nevertheless, the extent to which HPA-driven immunosuppression independently determines infection risk and long-term outcome remains incompletely resolved.

### 4.3 The parasympathetic nervous system

In addition to the sympathetic nervous system (SNS) and the hypothalamic-pituitary-adrenal (HPA) axis, the parasympathetic nervous system (PNS) has also been implicated in post-stroke immune modulation. For an extensive review on this topic refer to (71).

Experimental evidence indicates that parasympathetic activity is rapidly altered after stroke. Peripheral inflammatory mediators such as lipopolysaccharide (LPS), TNF-α, and IL-1β can activate afferent vagal pathways, transmitting immune signals to brainstem nuclei (71). This afferent signaling contributes to central integration of inflammatory inputs and may indirectly influence neuroendocrine responses, including activation of the HPA axis. In parallel, efferent vagal fibers release acetylcholine (ACh), the principal neurotransmitter of the parasympathetic system(See **Figure 1**).

This efferent arm constitutes the so-called cholinergic anti-inflammatory pathway, a neural reflex mechanism through which vagal signaling modulates immune function. Activation of α7 nicotinic acetylcholine receptors (α7nAChR) on macrophages suppresses the production of pro-inflammatory cytokines. *In vitro* studies have demonstrated that acetylcholine reduces TNF-α release from macrophages following LPS stimulation, supporting a direct immunomodulatory role of vagal signaling (72, 73).

The functional consequences of parasympathetic modulation after stroke appear to be context-dependent. In a rat model, sectioning parasympathetic nerve fibers innervating the circle of Willis aggravated ischemic injury, suggesting that central parasympathetic signaling may exert neuroprotective effects, potentially through modulation of cerebral blood flow or local inflammatory responses.

Conversely, in murine models, enhanced cholinergic signaling after stroke has been associated with suppression of pulmonary innate immune responses. Inhibition of this pathway, either by vagotomy or in α7 nicotinic acetylcholine receptor-deficient mice, restored pulmonary immune responsiveness and reduced the incidence of post-stroke pneumonia. These findings indicate that vagal cholinergic signaling contributes to systemic immunosuppression, particularly within the lung, and may facilitate susceptibility to infection (74).

In clinical studies, autonomic imbalance characterized by increased sympathetic activity and reduced parasympathetic tone has been associated with worse outcomes after acute ischemic stroke (71). However, the precise contribution of parasympathetic signaling to systemic immunosuppression in patients remains incompletely defined.

### 4.4 Additional and emerging mechanisms causing post-stroke immunosuppression

Sex hormones have also been implicated in peripheral post-stroke immunosuppression. A study by Offner and Hurn showed that estradiol (E2) replacement in mice subjected to experimental stroke reduced infarct size while ameliorating peripheral immunosuppression, supporting a protective role of E2 after stroke (75). In humans, however, a 2017 review concluded that clinical studies using exogenous estrogen have not demonstrated clear benefit regarding stroke risk improvement (76). Moreover, with the aging population and the increasing incidence of stroke, age must also be considered. Both male sex and older age are among the independent risk factors for stroke-associated pneumonia (18), the latter probably reflecting age-related immunosenescence (77).

Although activation of the sympathetic nervous system (SNS) and the hypothalamic-pituitary-adrenal (HPA) axis are the most extensively studied mechanisms underlying post-stroke immunosuppression, alternative pathways have also been proposed (See **Figure 1**). One of the earliest studies addressing this phenomenon was published by Halina Offner and colleagues, who described a profound lymphopenia affecting both spleen and thymus 96 hours after experimental stroke (78). This was associated with marked splenic atrophy and widespread apoptosis, particularly impacting B-cell populations (78). While the authors discussed the potential contribution of sympathetic signaling, they also proposed an additional mechanism: the induction and relative enrichment of CD4^+^CD25^+^ regulatory T cells. At 96 hours post-stroke, Tregs were significantly increased despite the overall loss of splenocytes, suggesting that these cells may be comparatively resistant to apoptosis and could actively contribute to the immunosuppressive state. Given that regulatory T cells are key modulators of immune homeostasis and are capable of suppressing effector T-cell and innate immune responses, their expansion represents a plausible additional pathway contributing to post-stroke immune dysfunction. Therefore, a conceivable complementary mechanism is the activation and relative dominance of Treg cells, which may counterbalance other lymphocyte-driven inflammatory pathways.

A similar conceptual framework was proposed by Emsley and Hopkins in their 2010 review (79), where post-stroke immunodepression is interpreted as a counter-regulatory response to the intense pro-inflammatory reaction triggered by cerebral ischemia. In this model, functional immunosuppression is viewed as a compensatory mechanism aimed at limiting inflammatory brain injury. This may involve a deviation from a Th1 to a Th2 immune profile, along with increased production of anti-inflammatory cytokines such as IL-10, ultimately dampening pro-inflammatory responses.

Additional mechanisms have been proposed over the years. In 2011, Denes and colleagues described stroke-induced changes in the bone marrow that point to a systemic hematopoietic reprogramming after cerebral ischemia (29). They showed that stroke triggers rapid activation of bone marrow myeloid cells, mobilization of CXCR2^+^ granulocytes, and a shift in cellular composition favoring the myeloid lineage. This process appears to involve both circulating inflammatory mediators and possible direct neuronal influences on the bone marrow. Although the authors did not observe bone marrow immunosuppression or intrinsic lymphopenia -in fact, bone marrow cells retained their capacity to respond to endotoxin stimulation- the shift toward a myeloid profile suggests a redistribution of hematopoietic resources. Such a bias could, at least in theory, influence peripheral lymphocyte availability and indirectly contribute to the later development of post-stroke immunosuppression.

More recent work identifying alternative pathways involved in post-stroke immunosuppression emerged nearly a decade later involving DAMPs, which are essential mediators of systemic inflammation after stroke. The initial release of DAMPs from the post-ischemic necrotic and apoptotic brain tissue led to activation of the cerebral endothelium and recruitment of local and circulating immune cells (80).

In 2018, Roth and colleagues proposed an additional mechanism of brain-periphery communication (81). Although their study primarily focused on stroke-accelerated atherosclerosis, they demonstrated that brain-derived alarmins-particularly HMGB1-are released into the circulation after stroke and activate systemic immune cells via RAGE signaling. Importantly, they showed that this alarmin-driven activation operates in parallel with, and not exclusively through, sympathetic signaling. While this study did not directly address peripheral immunosuppression, it provided mechanistic evidence that sterile danger signals (DAMPs) released from necrotic brain tissue can systemically reprogram the immune system.

Subsequent work from the same group in 2021 extended this concept and directly linked DAMP signaling to post-stroke lymphopenia (8). In that study, circulating cell-free double-stranded DNA (cfDNA) was identified as a key mediator of splenic T-cell loss. The authors demonstrated that cfDNA activates peripheral monocytes through the AIM2 inflammasome, leading to IL-1β production and the emergence of FasL-expressing monocytes. These FasL^+^ monocytes then induce extrinsic apoptosis of lymphocytes via the Fas-FasL pathway, providing a mechanistically defined route from tissue damage to systemic lymphopenia.

Moreover, other studies confirmed a general increase of cfDNA post-stroke in patients (80), and, particularly one study described this increased as early as 3h post-stroke (82). However, the origin of the circulating cfDNA remained unresolved. Addressing this question, more recent work from the group of Matthias Günther and Vikramjeet Singh provided evidence that stroke also affects gut-associated lymphoid tissue and identified neutrophils as a likely source of the circulating DNA (83). They showed that stroke induces the formation of neutrophil extracellular traps (NETs), which release extracellular DNA into the circulation. This NET-derived DNA was sufficient to drive lymphocyte depletion within Peyer’s patches, suggesting a mechanistic link between neutrophil activation, DAMP release, and intestinal immune suppression. Although the precise mechanism of neutrophil activation remains unclear, these findings connect neutrophil activation and NET formation to compartment-specific lymphocyte loss after stroke.

In summary, beyond activation of the SNS and HPA axis as classical drivers of post-stroke peripheral immunosuppression, several additional mechanisms have been brought into focus. These include counter-regulatory immune responses such as the expansion of regulatory T cells and a shift toward anti-inflammatory cytokine profiles, systemic hematopoietic reprogramming favoring myeloid over lymphoid lineages, and the release of damage-associated molecular patterns (DAMPs) from the injured brain, or potentially from secondary sources, that are capable of inducing lymphocyte depletion through defined molecular pathways. Whether these mechanisms operate independently, sequentially, or in a coordinated manner remains unclear. Likewise, their relative contribution to the overall immunosuppressive phenotype, and whether distinct mechanisms preferentially affect specific immune compartments or organs, are still unresolved questions. In the following section, we propose an integrative model that attempts to reconcile these pathways within a unified framework of post-stroke immune dysfunction.

## 5 Why does stroke induce peripheral immunosuppression?

Up to this point, we have summarized extensive evidence showing that stroke is followed by a state of peripheral immunosuppression, and we have reviewed the main mechanisms proposed to underlie this phenomenon. However, a more fundamental question remains unresolved: why does post-stroke immunosuppression occur in the first place? Is it an adaptive response aimed at protecting the injured brain from excessive inflammation? Is it a strategy to prevent immune recognition of newly exposed CNS antigens and the development of autoimmunity? Or is it simply a collateral consequence of disrupted neuroimmune regulation?

A dominant concept across multiple reviews is that peripheral immunosuppression may represent a brain-driven protective program (12, 17, 18, 79, 84). This view is consistent with abundant evidence showing that exaggerated inflammatory responses can promote secondary injury and exacerbate infarct evolution (85, 86). In this context, limiting lymphocyte migration into the ischemic brain and dampening systemic immune activation could theoretically reduce collateral inflammatory damage. From this perspective, stroke-induced immunosuppression may represent an evolutionary trade-off in which protection of the injured CNS is achieved at the expense of increased susceptibility to infection.

At the same time, inflammation is not intrinsically detrimental. In most organs, it is required to initiate tissue repair programs, and even in the adult brain, despite its limited regenerative capacity, immune responses contribute to debris clearance and resolution processes. However, there is not a lot of experimental data that can definitely state that immunosuppression is a purely protective adaptation from the brain itself.

A second, non-mutually exclusive possibility is that immunosuppression limits immune reactivity against CNS antigens released after ischemic injury. Under physiological conditions, immune exposure to many CNS antigens is restricted, in part due to the blood-brain barrier and other features of CNS immune privilege. Stroke disrupts these barriers and releases CNS-derived antigens into an inflammatory context, which could in principle favor the emergence of autoreactive immune responses. In line with this idea, experimental reversal of stroke-induced immunodepression by blocking stress axes (e.g., sympathetic and glucocorticoid signaling) increased CNS antigen-specific T-cell responses within the ischemic brain two weeks after MCAO, as reflected by a higher frequency of IFN-γ-producing cells upon stimulation with a myelin-derived peptide (67). Importantly, this increased CNS-directed reactivity was not mirrored by a clear systemic expansion of antigen-specific effector responses in the spleen, and it did not translate into observable EAE-like symptoms or worsened long-term functional outcome in that model. Additionally, blocking sympathetic nervous system reversed partially stroke-induce immunosuppression but did not aggravate functional outcome after experimental stroke in rats (87). These data support the notion that immunosuppression may contribute to restraining autoreactive responses in the injured CNS, but also suggest that enhancing CNS antigen-specific immunity does not necessarily result in clinically relevant autoimmune disease, at least under the conditions tested.

Finally, immunosuppression may reflect collateral dysregulation of neuroimmune control circuits. Over the last decades, it has become clear that the brain plays an active role in sensing and shaping systemic immune responses. Peripheral inflammation can be detected through humoral routes, particularly via circulating cytokines such as IL-1β, TNF-α, and IL-6 that reach specialized brain regions lacking a fully developed blood-brain barrier, including the circumventricular organs (CVOs) and the area postrema (see (88) for a detailed review on this topic). Cells within these structures express cytokine receptors and transduce peripheral inflammatory signals into central neuronal activation. For example, IL-1β signaling at the level of the organum vasculosum of the lamina terminalis (OVLT) and other CVOs activates hypothalamic and brainstem nuclei that subsequently engage autonomic and neuroendocrine pathways to modulate systemic immune activity.

In addition to humoral sensing, peripheral inflammation is also detected through neural pathways, particularly via vagal afferents that respond to inflammatory mediators and relay this information to the nucleus tractus solitarius (72, 73). This central integration enables activation of anti-inflammatory reflex circuits, such as the cholinergic anti-inflammatory pathway, which suppresses peripheral cytokine production through efferent vagal signaling. Through these coordinated humoral and neural mechanisms, the brain fine-tunes the magnitude and duration of systemic inflammatory responses, preventing excessive immune activation and limiting collateral tissue damage.

In the context of stroke, however, these regulatory circuits may become dysregulated. Because inflammation originates within the brain itself, the intensity and spatial distribution of inflammatory signals reaching central integrative structures may exceed those observed during peripheral inflammation. This could trigger an exaggerated counter-regulatory response, leading to systemic immunosuppression that is disproportionate to the actual peripheral infectious threat. Moreover, ischemic injury affecting key regulatory nuclei may directly impair neuroimmune homeostasis, resulting in maladaptive autonomic and neuroendocrine output.

Taken together, current evidence supports the idea that post-stroke immunosuppression may emerge from a combination of adaptive and maladaptive processes: limiting harmful neuroinflammation, restraining CNS antigen-specific immune activation, and/or reflecting dysregulated neuroimmune feedback circuits. Determining the relative weight of these drivers, and whether they differ across organs and time windows, remains a major challenge for the field. A useful way to interpret these apparently conflicting roles may therefore be to consider post-stroke immunosuppression as a context-dependent phenomenon, in which early limitation of excessive neuroinflammation could be beneficial, whereas sustained or compartment-specific peripheral immune suppression may become maladaptive by increasing susceptibility to infection.

## 6 Is it possible a different model of how stroke triggers post-stroke immunosuppression?

As outlined throughout this review, multiple mechanisms have been proposed to account for post-stroke immunosuppression, including activation of the sympathetic nervous system (SNS), stimulation of the hypothalamic-pituitary-adrenal (HPA) axis, engagement of parasympathetic pathways, release of damage-associated molecular patterns (DAMPs), hematopoietic reorganization within the bone marrow, and neutrophil activation. While several reviews have suggested that these pathways may operate in parallel, an important unresolved question remains: do these mechanisms function independently, or are they components of an interconnected regulatory network? Moreover, it is plausible that distinct pathways predominate in specific organs, such as spleen, bone marrow, lung, or gut-associated lymphoid tissue, resulting in a compartmentalized rather than uniform immunosuppressive phenotype.

**Figure 2:**
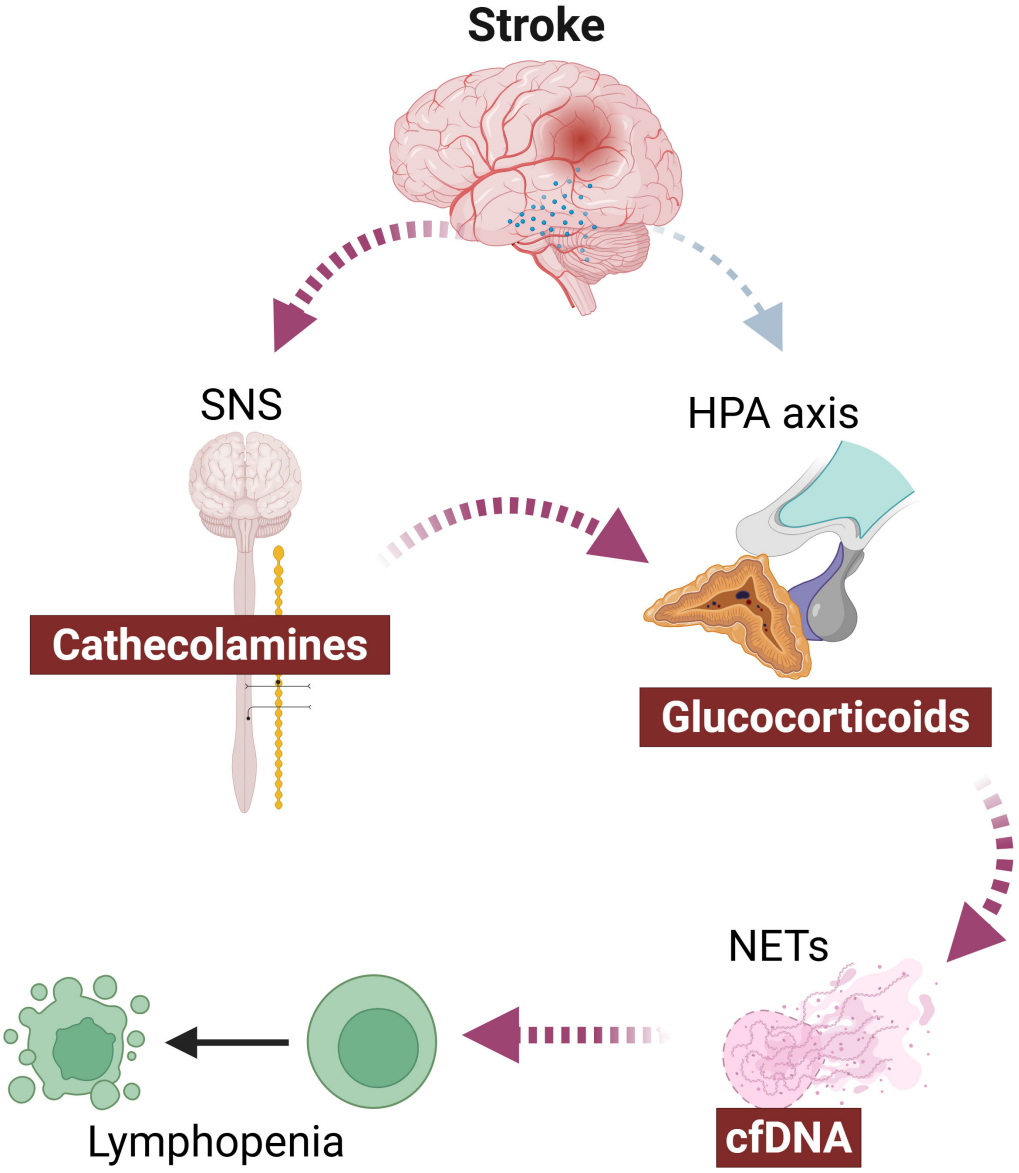
Beyond classical SNS and HPA activation: hypothesis for post-stroke immunosuppression mechanism. NETs, neutrophil extracellular traps; SNS, sympathetic nervous system.

One example of potential crosstalk is the interaction between the SNS and glucocorticoid release (See **Figure 2**). Under certain stress conditions, increased sympathetic tone has been shown to modulate adrenal glucocorticoid secretion (89). This phenomenon may be in agreement with observations that suggest that several routes for HPA-axis dysregulation are present early after stroke (90, 91). This interaction may help explain why both sympathectomy and pharmacological blockade of glucocorticoid signaling attenuate post-stroke immunosuppression in experimental models. Rather than representing entirely separate mechanisms, these interventions may target interconnected nodes within a shared neuroendocrine-immune network.

**Figure 3:**
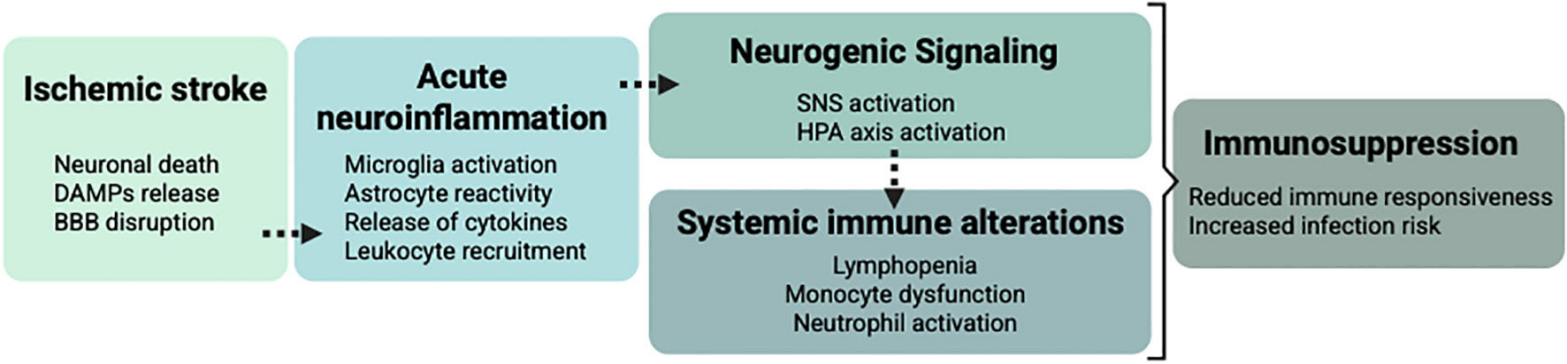
Sequential cascade from ischemic brain injury to systemic immunosuppression. BBB, blood-brain barrier; DAMPs, damage-associated molecular patterns; HPA, hypothalamic-pituitary adrenal; SNS, sympathetic nervous system.

Beyond classical SNS and HPA activation, intermediary mechanisms may further amplify immune dysregulation within the neurogenic–immune cascade following stroke (see **Figure 3**). In the context of chronic stress and cancer, glucocorticoids have been shown to promote neutrophil activation and the formation of neutrophil extracellular traps (NETs), a process implicated in metastatic progression (92). It is therefore plausible that the surge in glucocorticoids observed after stroke could similarly modulate neutrophil behavior, promoting NET formation as reported by Tuz et al (83). NET-derived extracellular DNA could then act as a DAMP, activating innate immune pathways that culminate in lymphocyte apoptosis and systemic immunosuppression as suggested by Roth et al (8) (See **Figures 2**, **3**).

This hypothesis is consistent with studies identifying circulating cell-free DNA as a mediator of post-stroke lymphopenia and others suggesting neutrophils and NETs as a potential source of that DNA. However, whether glucocorticoids are the primary trigger of neutrophil activation in stroke, or whether inflammatory or neuronal signals independently drive NETosis, remains unresolved. At present, this model integrates observations from different fields but lacks direct mechanistic confirmation in the stroke setting.

Taken together, these considerations suggest that post-stroke immunosuppression may arise either from multiple parallel pathways converging on a common phenotype or from interconnected mechanisms that reinforce one another. Such functional redundancy could explain why targeting distinct nodes, SNS signaling, glucocorticoid receptors, DAMP pathways, or neutrophil activation, has produced comparable protective effects in experimental models. A deeper understanding of this mechanistic heterogeneity will be essential for designing effective strategies to prevent post-stroke immunosuppression.

## 7 Discussion

Stroke remains a major cause of mortality, long-term disability, and profound psychosocial burden for both patients and their families. Despite significant advances in reperfusion strategies, including thrombolysis and mechanical thrombectomy, a substantial proportion of patients remain ineligible for these interventions or do not receive them in time. Beyond the hyperacute phase, stroke survivors frequently develop secondary complications that further impair recovery and quality of life. Among these, post-stroke immunosuppression represents a particularly relevant and yet incompletely understood phenomenon.

Although immune dysfunction after acute injury is not exclusive to stroke, the mechanisms underlying post-stroke immunosuppression remain debated. Both the “how” and the “why” are still unresolved. Is immunosuppression an adaptive evolutionary mechanism through which the injured brain attempts to protect itself from excessive inflammation or autoimmunity? Or is it a collateral consequence of disrupted neuroimmune homeostasis, reflecting maladaptive dysregulation of stress and inflammatory circuits? Regardless of its origin, what appears clear is that post-stroke immunosuppression compromises normal immune function. This impairment is strongly associated with increased susceptibility to infections and poorer clinical outcomes.

Understanding this phenomenon at a mechanistic level is not merely an academic exercise; it has direct therapeutic implications. If post-stroke immunosuppression serves a protective function by limiting secondary neuroinflammation or restraining autoreactive immune responses, indiscriminate reversal of this state could carry unintended consequences. Conversely, if immunosuppression largely represents a bystander effect of neuroendocrine activation without clear benefit to the injured brain, then more aggressive strategies to restore peripheral immune competence may be justified to prevent infectious complications.

An alternative and potentially more refined strategy may involve dissociating central and peripheral immune modulation-preserving mechanisms that limit detrimental neuroinflammation within the brain while simultaneously protecting the periphery from infection-related morbidity. Achieving such precision will require a deeper and more integrated understanding of the temporal, spatial, and mechanistic heterogeneity of post-stroke immune responses.

In this review, we have attempted to synthesize current knowledge and propose a framework that integrates seemingly disparate mechanisms into a coherent working model. However, further experimental and translational studies are needed to dissect these pathways with greater resolution and to determine which components are adaptive, maladaptive, or context-dependent. Only through such detailed mechanistic clarification will it be possible to rationally design therapeutic strategies that mitigate complications without compromising endogenous protective programs.
